# baysc: An R package for Bayesian survey clustering

**DOI:** 10.21105/joss.08382

**Published:** 2026-03-11

**Authors:** Stephanie M. Wu, Matthew R. Williams, Terrance D. Savitsky, Briana J. K. Stephenson

**Affiliations:** 1 Division of Psychiatry, UCL, London, U.K.; 2 RTI International, Research Triangle Park, North Carolina, U.S.A; 3 Office of Survey Methods Research, U.S. Bureau of Labor Statistics, Washington, DC, U.S.A; 4 Department of Biostatistics, Harvard T.H. Chan School of Public Health, Boston, Massachusetts, U.S.A

## Summary

Model-based clustering methods allow a large number of correlated variables to be summarized into underlying patterns, where each pattern describes a cluster and each individual is assigned to a cluster. Example applications include identifying dietary patterns from dietary intake data (Stephenson et al., 2020) and creating profiles of health and development among children (Lanza & Cooper, 2016). Bayesian formulations of such analyses allow the number of clusters to be determined by the data rather than through researcher post-hoc analyses.

Interest may also lie in relating the identified clusters to an outcome, either through a secondary regression step or an all-in-one “supervised” approach that uses information from the outcome to inform the clustering process. When such clustering methods are applied to survey data, failure to account for the complex survey design and incorporate survey weights into the estimation leads to biased estimation and inference when the results are generalized to the population outside of the survey data.

The baysc R package implements the methods proposed in Stephenson, Wu, & Dominici (2023) and Wu, Williams, Savitsky, & Stephenson (2024), providing functionality to allow for Bayesian clustering analyses – both unsupervised and supervised – to be performed while incorporating survey weights and design features that account for complex survey sampling designs. Asymptotically correct point estimates and credible intervals are produced with respect to the underlying population from which the observed sample was generated. This novel feature allows for application of latent class analysis (LCA) to datasets realized from surveys administered by government statistical agencies. The package uses methods derived from the LCA literature and focuses on clustering in the setting where the correlated variables are categorical and the outcome, where applicable, is binary. The package includes additional functions for plotting and summarizing output, as well as an example dataset from the National Health and Nutrition Examination Survey (NHANES) containing dietary intake and hypertension data among low-income women in the United States (National Center for Health Statistics, 2023).

Detailed information regarding package usage and functionality can be found in the vignette titled, “An introduction to the baysc package,” available on the package GitHub repo located at https://github.com/smwu/baysc/.

## Statement of Need

A number of R packages provide functionality for model-based clustering in R. Frequentist approaches include poLCA (Linzer & Lewis, 2011) for classical LCA, randomLCA (Beath, 2017) for LCA with individual-specific random effects, and mclust (Scrucca, Fraley, Murphy, & Raftery, 2023) and tidyLPA (Rosenberg, Beymer, Anderson, Van Lissa, & Schmidt, 2019) for clustering of continuous variables. BayesLCA (White & Murphy, 2014) and BayesBinMix (Papastamoulis & Rattray, 2017) use Bayesian approaches for categorical and binary data, respectively. PReMiuM (Liverani, Hastie, Azizi, Papathomas, & Richardson, 2015) fits a wide variety of supervised models that handle various types of discrete and continuous exposure and outcome data. However, these packages do not allow for survey weights and complex survey design to be incorporated to ensure valid estimation and inference when using survey data.

The baysc package implements a weighted pseudo-likelihood approach proposed in Stephenson et al. (2023) and Wu et al. (2024) that can integrate survey sampling weights when performing cluster analysis using categorical or binary data. The models adjust for stratification, clustering, and informative sampling to provide accurate point and variance estimation. When interest lies in how clusters are related to additional variables, such as a binary outcome, users can either: 1) adopt a two-step approach where unsupervised clustering is performed and then a secondary regression is fit, or 2) use the all-in-one supervised approach that jointly models all variables, precisely propagating measurement error from the clustering analysis into the outcome regression model. The supervised approach also uses a mixture reference coding scheme to allow interaction effects between the clusters and the outcome to be captured. Statistical details can be found in the vignette, “An introduction to the baysc package,” and Wu et al. (2024).

## Data Availability

Review Repository Archive
